# Immune Dysregulation After COVID-19: Longitudinal Analysis up to 9 Months

**DOI:** 10.3390/ijms27115137

**Published:** 2026-06-05

**Authors:** Paweł Mitkowski, Monika Leśniak, Dagmara Kobza, Karolina Aleksandrowicz, Aleksander Chodowiec, Krzysztof Piwowarek, Wojciech Włodarczyk, Klaudia Porębska, Katarzyna Plewka-Barcik, Agata Borkowska, Renata Rożyńska, Ewa Pietruszka-Wałęka, Andrzej Wojtyszek, Karina Jahnz-Różyk, Marcin Pękalski, Andrzej Chciałowski, Robert Zdanowski, Krzysztof Kłos

**Affiliations:** 1 Laboratory of Molecular Oncology and Innovative Therapies, Military Institute of Medicine–National Research Institute, 04-141 Warsaw, Poland; pmitkowski@wim.mil.pl (P.M.); mlesniak@wim.mil.pl (M.L.); dkobza@wim.mil.pl (D.K.); kaleksandrowicz@wim.mil.pl (K.A.); achodowiec@wim.mil.pl (A.C.); kpiwowarek@wim.mil.pl (K.P.); kporebska@wim.mil.pl (K.P.); aborkowska@wim.mil.pl (A.B.); mpekalski@wim.mil.pl (M.P.); 2 BioMedChem Doctoral School of the University of Lodz and Lodz Institutes of the Polish Academy of Sciences, University of Lodz, 90-136 Lodz, Poland; 3 Department of Internal Medicine, Infectious Diseases and Allergology, Military Institute of Medicine–National Research Institute, 04-141 Warsaw, Poland; wwlodarczyk@wim.mil.pl (W.W.); kplewka@wim.mil.pl (K.P.-B.); achcialowski@wim.mil.pl (A.C.); kklos@wim.mil.pl (K.K.); 4 Department of Internal Medicine, Allergology, Pneumonology and Clinical Immunology, Military Institute of Medicine–National Research Institute, 04-141 Warsaw, Poland; rrozynska@wim.mil.pl (R.R.); epietruszka@wim.mil.pl (E.P.-W.); kjrozyk@wim.mil.pl (K.J.-R.); 5 7th Polish Navy Hospital, 80-308 Gdansk, Poland; a.wojtyszek@7szmw.pl; 6 JDRF/Wellcome Diabetes and Inflammation Laboratory, Wellcome Centre for Human Genetics, University of Oxford, Oxford OX1 2JD, UK

**Keywords:** COVID-19, immune dysregulation, biomarkers, cytokine storm, disease severity

## Abstract

SARS-CoV-2 infection triggers a strong inflammatory response, and persistent symptoms after recovery are thought to reflect prolonged systemic dysregulation. However, mechanisms underlying long COVID remain unclear. This study evaluated longitudinal changes in cytokine hematological and biochemical parameters up to nine months after hospitalization for COVID-19 and examined their relationship with persistent symptoms, vaccination status, and the occurrence of comorbidities. Long COVID patients showed sustained elevations in IL-2, IL-6, IL-10, IL-17A, CXCL9, TNF-α, and IFN-γ compared with healthy controls, exhibiting heterogeneous temporal trajectories. Specifically, levels of IL-2, IL-10, TNF-α, and creatinine continued to increase over time, whereas IFN-γ, LDH, CCL2, CXCL9, and CXCL10 declined. Other parameters exhibited greater variability without a uniform longitudinal pattern. IL-6 demonstrated consistently high diagnostic performance in distinguishing individuals after COVID-19 from healthy controls (AUC > 0.82). Aging substantially affects cytokine profiles and systemic inflammatory status; therefore, residual age-related confounding cannot be fully excluded despite statistical adjustment. Although symptoms such as fatigue, cough, and dyspnea were still reported at nine months, no stable associations were identified between cytokine concentrations and clinical manifestations. These findings indicate that while immune alterations persist long after acute infection, systemic cytokine measurements alone may be insufficient to explain the presence or persistence of symptoms. The results suggest that long COVID reflects multifactorial processes extending beyond systemic inflammation and highlight the need for further research into mechanisms driving long-term long COVID sequelae.

## 1. Introduction

COVID-19 is a respiratory disease caused by infection with severe acute respiratory syndrome coronavirus 2 (SARS-CoV-2), an enveloped, positive-sense, single-stranded RNA virus of the Coronaviridae family [1]. While many infections are asymptomatic or mild, a clinically meaningful fraction progresses to pneumonia requiring hospitalization, and approximately 10% of patients with pneumonia require intensive care unit (ICU) admission with or without mechanical ventilation [2].

In COVID-19, the cytokine storm denotes a state of excessive cytokine and chemokine production that disrupts immune homeostasis and drives inflammatory cascades [3]. In severe disease, it is considered a major contributor to clinical deterioration, supported by studies linking disease progression and severity with elevated circulating levels of pro-inflammatory mediators [4,5,6]. In critical COVID-19, this hyperinflammatory response contributes to the development of acute respiratory distress syndrome (ARDS), endothelial injury, and disruption of alveolar integrity [3,7]. Interestingly, other respiratory viruses induce distinct cytokine patterns, but the key determinant of severity is whether the host can effectively resolve the pro-inflammatory response. SARS-CoV and MERS-CoV trigger cytokine release syndromes that often fail to resolve. In contrast, influenza A viruses induce strong cytokine storms but retain effective resolution mechanisms. SARS-CoV-2 exhibits an intermediate profile: in most patients, inflammation is eventually downregulated, although a subset shows persistent dysregulation [8].

Beyond the acute infection, many individuals experience persistent or newly emerging symptoms after apparent recovery. This syndrome has been referred to as long COVID, post-acute sequelae of COVID-19 (PASC), or post-COVID-19 condition. In the present study, we use the term long COVID to refer to this condition. The World Health Organization (WHO) defines the long COVID condition as a state that occurs in people with a history of probable or confirmed SARS-CoV-2 infection, which usually manifests three months after symptom onset, persists for at least two months, and cannot be explained by any other diagnosis [9]. In addition to symptom burdens, including cardiorespiratory complaints, systemic manifestation (particularly fatigue), and neurological features [10], nationwide population-based research from Norway confirmed that long COVID condition was correlated with elevated risks of thrombocytopenia, agranulocytosis, and selected neurological symptoms [11].

A large meta-analysis (144 studies) reported a global pooled prevalence of long COVID of 36%, reaching 51% in South America. The prevalence persisted over time, with 35% at <1 year of follow-up and 46% at 1–2 years [12]. This may partly reflect delayed symptom recognition rather than a true increase in incidence over time.

Long COVID is associated with persistent immune dysregulation involving not only cytokines or chemokines, but also T cells, NK cells, and monocytes [13], as well as metabolic and lipid disturbances accompanying chronic inflammation [14]. The long COVID syndrome is widely recognized as physiologically diverse, with at least two phenotypes found using proteomic and immunological profiling. The first is an inflammatory subtype characterized by chronic systemic inflammation, enhanced neutrophil activation, dysregulated B-cells, autoreactivity signatures and higher cytokines and chemokines detectable months after acute infection [15]. The second category is non-inflammatory, with symptoms persisting even in the absence of prolonged immune activation and molecular profiles similar to those observed in healthy individuals [15]. Importantly, these immune alterations occur alongside changes in hematological and coagulation parameters, indicating that long COVID reflects a broader multisystem disturbance rather than an isolated cytokine-driven process.

These observations place immune dysregulation at the center of both severe acute COVID-19 and a substantial fraction of long COVID. However, key questions remain unresolved, particularly which inflammatory mediators remain altered months after discharge in cohorts with clinically significant disease requiring hospitalization and whether post-acute immune profiles reflect initial disease severity or relate to residual symptoms [16]. Several long COVID studies have shown that blood cytokine patterns can remain different from those in healthy individuals and may relate to the severity of a patient’s symptoms [17,18,19]. In addition, some cohorts show signs of persistent low-grade inflammation, together with changes in leukocyte profiles, in at least a subset of individuals [20,21]. These findings suggest that recovery after COVID-19 supports repeated and longitudinal measurements to distinguish short-lived inflammatory changes from dysregulation that persists or evolves over time.

In this study, we performed a longitudinal assessment of serum cytokines and chemokines, with additional analysis of biochemical and hematological parameters in adults hospitalized for moderate or severe COVID-19, with follow-up sampling at 3, 6, and 9 months after hospital discharge. A key knowledge gap remains the lack of longitudinal studies that simultaneously assess cytokines, chemokines and routine biochemical and hematological parameters across multiple post-acute time points in comparison with healthy individuals. By addressing this gap, our study provides an integrated view of immune and laboratory alterations over time and may help improve the understanding of the complex, multifactorial processes underlying long COVID.

## 2. Results

### 2.1. Characteristics of the Study Population

Among all recruited COVID-19 patients, 116 (79.9%) reported at least one comorbidity. The most common conditions were hypertension (78 patients, 56.1%), overweight (34 patients, 24.5%), and type 2 diabetes (31 patients, 22.3%). Additional comorbidities are summarized in Table 1. The sample was collected during successive waves of the COVID-19 pandemic, when the Delta variant was predominant (March–May 2021) [22]. In Poland, the epidemic was marked by pronounced fluctuations in case numbers and mortality, with the country periodically ranking among those with the highest COVID-19 death rates in Europe [22].

### 2.2. Cytokine Profiles According to COVID-19 Severity

**Figure 1:**
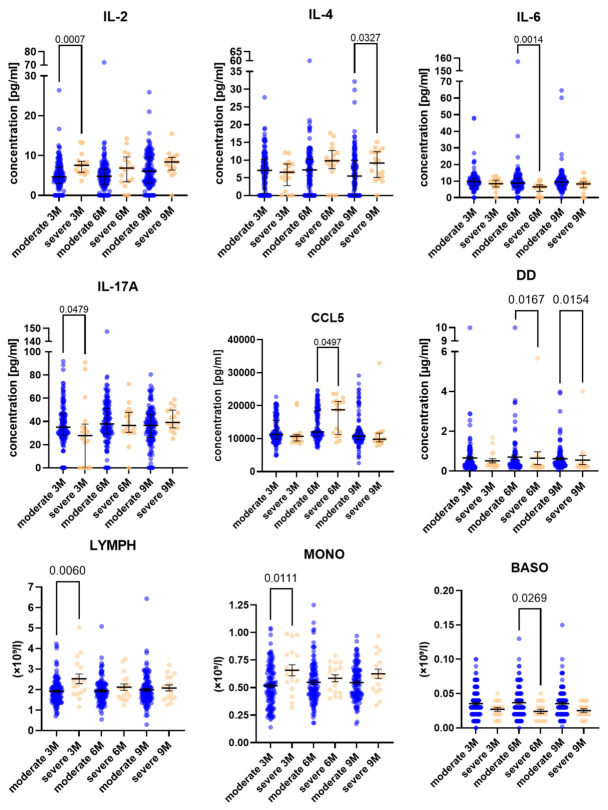
Standard inflammatory, biochemical and hematological parameters in patients with moderate or severe COVID-19 across follow-up time points. Individuals with moderate COVID-19 are shown in blue, with severe COVID-19 in orange. Data are shown as median with interquartile range; additionally, all data points for each individual are shown. Statistical analyses were performed using the two-tailed Mann–Whitney U test.

Cytokine levels and the occurrence of symptoms (cough, dyspnea, fatigue), as well as standard inflammatory, biochemical and hematological parameters, were compared at each time point. Data distribution was not normal; therefore, group comparisons were performed using the Mann–Whitney U test (Table S1). Statistically significant increases in the severe group were observed for IL-2 (*p* = 0.0007), lymphocyte counts (*p* = 0.0076), and monocyte counts (*p* = 0.0118) at 3 months; for CCL5 (*p* = 0.0497) at 6 months; and for IL-4 (*p* = 0.0327) at 9 months. Significant decreases in the severe group were observed for IL-17A (*p* = 0.0479) at 3 months; for IL-6 (*p* = 0.0014) and basophil counts (*p* = 0.0269) at 6 months; and for D-dimers at 3 and 9 months (*p* = 0.0167 and *p* = 0.0154, respectively) (Figure 1). No other parameters demonstrated severity-dependent differences (Figure S1). Overall, only limited differences between severity groups were observed, suggesting that long-term immune profiles are not strongly determined by initial disease severity.

Additionally, neither symptom occurrence nor the presence of comorbidities was significantly associated with disease severity (*p* > 0.05; Figure S2). However, comorbidities were associated with a higher frequency of symptoms at 3 months (*p* = 0.029), an effect that was not observed at later time points (Figure S3a). When stratified by comorbidity type, metabolic conditions (diabetes mellitus and abdominal obesity) were associated with a significantly higher prevalence of symptoms only at 3 months (*p* = 0.042) (Figure S3b). In contrast, no significant association between cardiovascular comorbidities (hypertension, coronary artery disease, chronic heart failure, atrial fibrillation, or other arrhythmias) and symptom occurrence was observed at any time point (Figure S3c).

The association between symptom occurrence and cytokine levels was also assessed. No significant differences were observed at 3 months. However, at 6 months, higher levels of IL-17A (*p* = 0.0167), IgM (*p* = 0.0056), and platelet counts (*p* = 0.0022) were associated with symptom persistence. At 9 months, patients with symptoms exhibited higher levels of platelets (*p* = 0.0185), IgM (*p* = 0.0322), PCT (*p* = 0.0281), and white blood cell counts (*p* = 0.0228) (Table S2A).

### 2.3. Biomarker Profiles Compared to Healthy Controls

**Figure 2:**
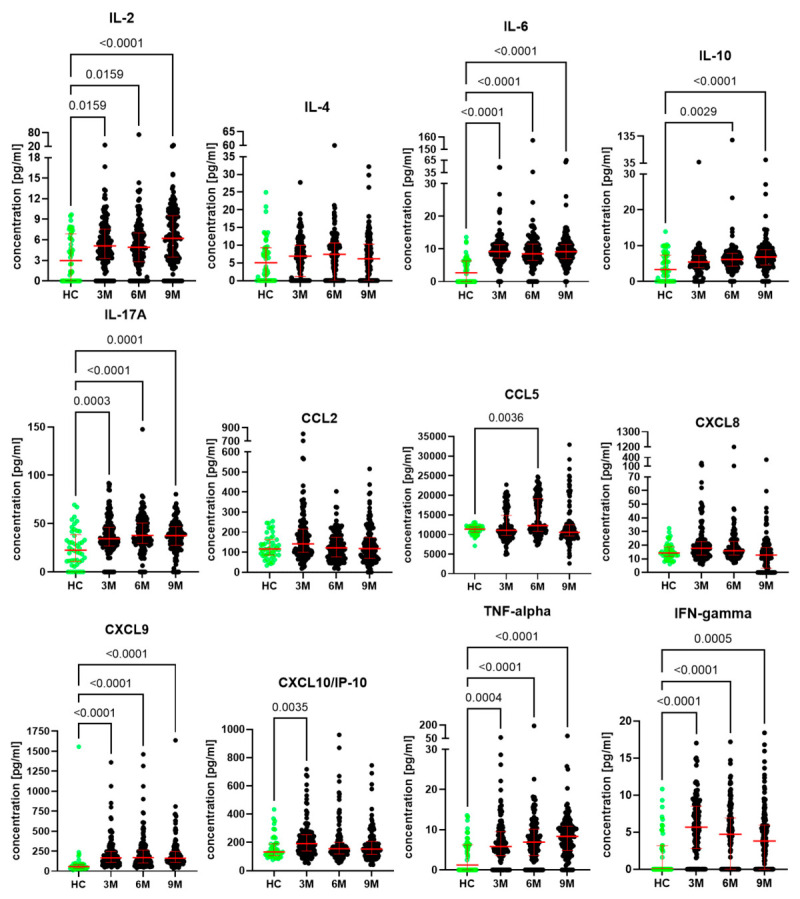
Comparison of cytokine concentration between groups at 3, 6, and 9 months after COVID-19 (3M, 6M, 9M, respectively) with healthy controls. Data are shown as median values and interquartile range indicated (red lines); additionally, all data points for each individual are shown. Healthy control is shown in green, and long COVID in black. Statistical analyses were performed using the Kruskal–Wallis test followed by the Benjamini–Hochberg false discovery rate correction.

Long COVID patients exhibited abnormalities in cytokine profiles compared with HCs (Figure 2). Concentrations of IL-2 (3M *p* = 0.0159, 6M *p* = 0.0159, 9M *p* < 0.0001), IL-6 (all *p* < 0.0001), IL-17A (3M *p* = 0.0003, 6M and 9M *p* < 0.0001), CXCL9 (*p* < 0.0001), TNF-α (*p* < 0.0001), and IFN-γ (3M and 6M *p* < 0.0001, 9M *p* = 0.0005) were significantly elevated in long COVID patients relative to HCs at each time point. CXCL10 (*p* = 0.0035) showed a transient increase at 3 months, CCL5 (*p* = 0.0036) at 6 months, and IL 10 at 3 (*p* = 0.0029) and 6 months (*p* < 0.0001) compared to HC levels. CXCL8, CCL2, and IL-4 did not differ significantly between the groups.

### 2.4. Impact of Comorbidities on Biomarker Profiles

In the overall analysis assessing the impact of comorbidities on cytokine levels, their presence was associated with significantly elevated levels of CXCL8 (*p* = 0.0263), CXCL9 (*p* = 0.00366), IL-2 (*p* = 0.0461), and monocyte percentage (*p* = 0.0168) at 3 months. At 6 months, CXCL8 remained significantly elevated (*p* = 0.0002), while basophil levels were reduced (*p* = 0.022). At 9 months, basophil levels remained decreased (*p* = 0.0492), along with IFN-γ (*p* = 0.0443), IgG levels (*p* = 0.0145), and platelet counts (*p* = 0.0145) (Table S2B).

In subgroup analyses, cardiovascular comorbidities were associated with higher levels of CXCL9 (*p* = 0.0004) and IL-6 (*p* = 0.0045), as well as lower eGFR (*p* = 0.0486), at 3 months. Additionally, transient changes were observed, including elevated CXCL8 at 6 months (*p* = 0.0005) and increased IL-2 levels (*p* = 0.0126), basophil levels (*p* = 0.0196), and eosinophil percentage (*p* = 0.0177) at 9 months (Table S2C).

In patients with metabolic comorbidities, decreased eGFR was consistently observed across all time points (3, 6, and 9 months), while CRP was elevated at 3 and 6 months and CXCL9 at 6 and 9 months. At 3 months, higher eosinophil counts (*p* = 0.0235) and CXCL10 (*p* = 0.0202) and lower AST levels (*p* = 0.0361) were observed. At 6 months, higher CXCL8 levels (*p* = 0.0065) were noted. At 9 months, higher IL-2 levels (*p* = 0.0046), and lower IL-4 levels (*p* = 0.0343) were observed (Table S2D).

### 2.5. Vaccination

**Figure 3:**
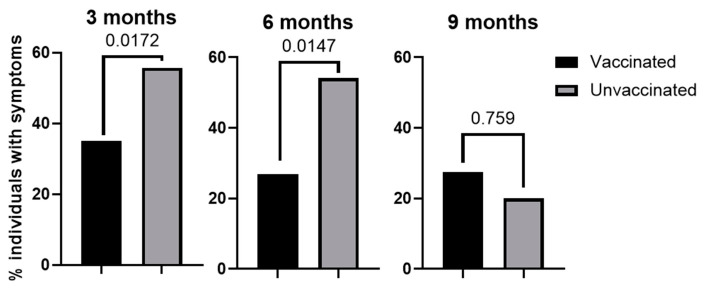
Comparison of the occurrence of at least one symptom between vaccinated and unvaccinated groups.

Differences in laboratory parameters and cytokine levels were compared between vaccinated once, twice and unvaccinated individuals at individual time points. As expected, only IgG concentrations were consistently higher in vaccinated individuals, particularly in those who received two doses. Additionally, only IL-4 showed differences across follow-up (Table S2E). However, the presence of at least one symptom was significantly less frequent among vaccinated individuals at 3 and 6 months (3 months: 35.0% vs. 55.7%, *p* = 0.0172; 6 months: 26.9% vs. 54.2%, *p* = 0.0147); no difference was observed at 9 months (27.4% vs. 20.0%, *p* = 0.759) (Figure 3).

### 2.6. Longitudinal Dynamics of Biomarker Levels

**Figure 4:**
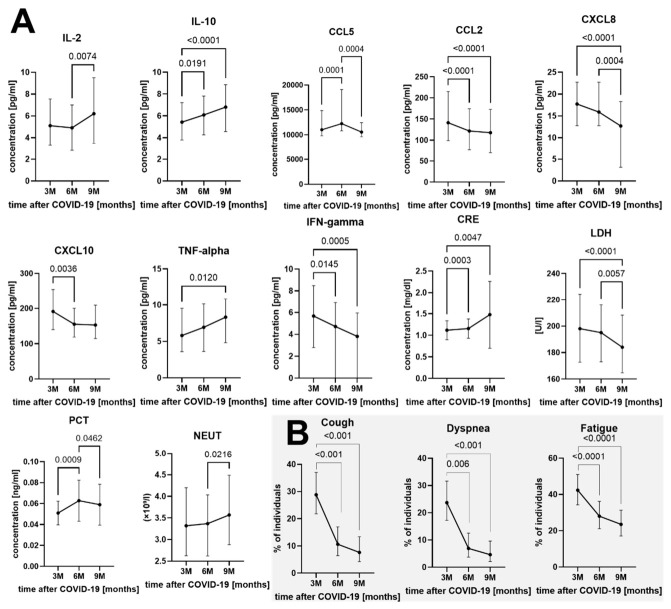
Longitudinal changes in inflammatory, biochemical and hematological parameters in long COVID individuals, at 3, 6, and 9 months after infection. (**A**) Data are shown as median with interquartile range. For CRE and PCT, data are shown as mean with 95% confidential interval. Statistical analyses were performed using the Friedman test with false discovery rate. (**B**) Data are shown as percent of individuals with symptoms, with 95% confidence intervals calculated using the Wilson score method. Statistical analyses were performed using Cochran’s Q test with McNemar’s post hoc test with Bonferroni correction.

Longitudinal analysis revealed distinct temporal patterns among the analyzed parameters (Figure 4A). IL-10 concentrations increased significantly over time, with differences observed between 3 and 6 months (*p* = 0.0191) and between 3 and 9 months (*p* < 0.0001). TNF-α also showed a progressive increase, reaching significance between 3 and 9 months (*p* = 0.0120). Similarly, IL-2 levels rose significantly between 6 and 9 months (*p* = 0.0074). In all cases, these changes resulted in increasing divergence from HCs. Conversely, IFN-γ showed a significant decrease between 3 and 6 months (*p* = 0.0145) and between 3 and 9 months (*p* = 0.0005). CCL2 also declined significantly between 3 and 6 and 3 and 9 months (both *p* < 0.0001). CXCL8 concentrations decreased between 3 and 6 months (*p* < 0.0001) and further between 6 and 9 months (*p* = 0.0004), approaching levels observed in HCs. CCL5 displayed a transient peak at 6 months, with a significant increase between 3 and 6 months (*p* = 0.0001), followed by a decrease between 6 and 9 months (*p* = 0.0004). Conversely, CXCL10 concentrations declined after 3 months, with a significant decrease between 3 and 6 months (*p* = 0.0036), and remained stable thereafter. Creatinine levels increased significantly between 3 and 6 months (*p* = 0.0003) and between 3 and 9 months (*p* = 0.0047), while neutrophil counts rose between 6 and 9 months (*p* = 0.0120). Procalcitonin displayed a transient peak at 6 months, increasing between 3 and 6 months (*p* = 0.0009) and decreasing between 6 and 9 months (*p* = 0.0462). In contrast, lactate dehydrogenase decreased progressively, with significant reductions between 3 and 9 months (*p* < 0.0001) and between 6 and 9 months (*p* = 0.00657). For IL-4, IL-6, IL-17A, and CXCL9, no significant changes in concentrations over time were observed (Figure S4).

A significant decline in the prevalence of cough, fatigue, and dyspnea was observed over time (all *p* < 0.001). The most pronounced reduction in symptom occurrence occurred between 3 and 6 months after infection. Post hoc analysis confirmed significant decreases between 3 and 6 months and between 3 and 9 months for all analyzed symptoms, while no significant differences were observed between 6 and 9 months, indicating a plateau in symptom resolution. Despite the overall improvement, a subset of patients continued to report persistent symptoms at 9 months, with fatigue being the most common (23%), followed by cough (8%) and dyspnea (5%) (Figure 4B).

### 2.7. Diagnostics

ROC analysis was performed to evaluate the ability of individual variables to discriminate long COVID patients from HCs (Table S3). IL-6 and CXCL9 demonstrated good diagnostic accuracy (AUC > 0.82); however, age individually showed strong discriminatory performance (AUC 0.96), indicating a potential confounding effect (Table 2). To account for this, age-adjusted multivariable logistic regression confirmed that only IL-6 remained significantly associated with long COVID status, whereas the effect of CXCL9 was attenuated, suggesting that its discriminatory performance was largely driven by age (Table S4A). To reduce the impact of age on the model, an additional analysis was performed in a subgroup of long COVID participants aged 30–50 years. This resulted in a decrease in the AUC for age to 0.77, while the diagnostic performance of the cytokines remained largely unchanged. Consistent with previous analyses, multivariable logistic regression adjusted for age did not materially change the effect estimates for IL-6, indicating that its discriminatory performance was not driven by age differences. In contrast, CXCL9 odds ratios suggested that its discriminatory ability was largely attributable to age (Table S4B).

### 2.8. Correlation Analysis

Spearman correlation analysis revealed an association between age and CXCL9 levels at 3 months (ρ = 0.49), which subsequently disappeared, dropping below 0.2 at later time points. Notably, no cytokine–cytokine correlations were observed in the long COVID group, including the IL-6–CXCL9 pair, whereas HCs exhibited more numerous and stable inter-marker relationships (Table 3, Table S5). This finding suggests a loss of coordinated immune regulation following COVID-19.

## 3. Discussion

In our previous analysis of this cohort (Kłos et al., 2024) [23], which included functional and imaging assessment of the respiratory system, we demonstrated that patients recovering from severe COVID-19 exhibited reduced lung volumes, features of parenchymal fibrosis, and post-inflammatory small-airway narrowing undetectable on computed tomography. These abnormalities may contribute to the persistence of respiratory symptoms [23]. The present study extends those observations by examining long-term systemic biomarker monitoring.

We observed a significant decline in the prevalence of respiratory symptoms over time, with the most pronounced improvement occurring within the first 6 months after infection; however, some symptoms were persistent at 9 months, particularly fatigue. This temporal pattern is consistent with previous reports showing an initial dynamic recovery phase followed by a slower resolution, with a substantial proportion of patients remaining symptomatic up to 2 years after infection [24]. It should be noted that our analysis focused primarily on respiratory symptoms, whereas other studies have reported a broader spectrum of long-term manifestations, including musculoskeletal, cardiovascular, and gastrointestinal symptoms, which may explain the higher overall prevalence of long COVID described in the literature [24].

Immunological differences between moderate and severe disease are pronounced during the acute phase but diminish during recovery [25]. Early convalescence is characterized by predominant pro-inflammatory signaling that gradually shifts toward tissue-repair pathways; however, no stable immunological pattern reliably differentiates the severity of prior disease [26].

Although our study did not assess biomarkers of acute disease severity, it is worth noting that numerous reports from the acute phase of COVID-19 have linked severe disease and mortality to increased concentrations of pro-inflammatory cytokines and chemokines. For example, meta-analytic evidence from 2022 indicates that acute COVID-19 severity is associated mainly with increased circulating levels of IL-6, IL-10, TNF-α, IL-1β, IL-8, IL-17, and IL-2R [27].

Overall, our findings are largely consistent with the available literature, including meta-analytic evidence [28], indicating persistent immune activation following COVID-19. The majority of cytokines identified as elevated in previous studies, such as IL-6, IL-2, TNF-α, IFN-γ, and CXCL10, showed similar patterns in our cohort, supporting the presence of sustained inflammatory signaling [28,29]. At the same time, considerable heterogeneity across studies is evident, particularly for chemokines such as CCL2, CCL5 and CXCL10, which appear to be more variable and context-dependent [28,29]. In line with this, while some markers in our study followed previously reported trajectories, others, including the transient peak of CCL5 and the early elevation followed by the normalization of CXCL10, were not consistently observed in other cohorts [29]. These discrepancies likely reflect differences in study populations, disease severity, and definitions of long COVID conditions, and suggest that certain cytokines may represent nonspecific or dynamic markers rather than stable features of long COVID dysregulation [21].

A clear example of the impact of long COVID definition on observed immune profiles is provided by previous studies in which symptom-based assessment yielded results opposite to those obtained using objective measures [30]. When long COVID was defined using online questionnaires, IL-6, IL-10, IL-17A and TNF-α were reduced at 3 months compared to HCs and normalized by 6 months, with a concurrent increase in IL-10. In contrast, in our cohort these cytokines remained elevated. Similarly, CXCL10 did not differ from controls in that study, whereas in our data it was elevated at 3 months and subsequently declined to levels comparable to HCs. Notably, when long COVID was defined based on impaired pulmonary function (diffusing capacity of the lung for carbon monoxide), IL-6, TNF-α, IL-17A, IL-10 and CXCL10 were elevated, consistent with the sustained inflammatory profile observed in our study [30].

In our long COVID cohort, only sparse correlations between cytokines were observed, whereas healthy individuals exhibited more inter-cytokine associations. This pattern suggests a disrupted regulatory cytokine network in individuals recovering from COVID-19, with preserved physiological interdependencies in HCs. In contrast to some reports suggesting associations between cytokine levels and persistent symptoms, we did not observe a clear relationship between inflammatory markers and clinical manifestations. Long COVID is increasingly recognized as a heterogeneous and multisystem disorder. The absence of significant parameter–symptom correlations may reflect several factors, including the limited cytokine panel assessed, the dynamic and potentially compartmentalized nature of immune responses, the timing of sample collection, and the substantial biological heterogeneity of post-COVID-19 phenotypes [31,32]. Previous studies demonstrated that integrated biomarker approaches combining inflammatory and non-inflammatory parameters, including lymphocyte/monocyte ratios, D-dimer levels, ferritin, and iron-related markers, may provide a more comprehensive view into COVID-19 pathophysiology than isolated cytokine measurements alone [33]. Accordingly, our findings support the view that circulating cytokines represent only one aspect of the complex biological processes underlying long COVID, and that the relationship between immune activation and symptom burden remains incompletely understood [21,34,35].

Age is an important determinant of baseline inflammatory and cytokine profiles, which may influence the observed differences between long COVID individuals and HCs [36,37]. Consistent with our analyses, adjusting for age did not materially change the effect estimates for IL-6, indicating that its discriminatory performance was not driven solely by age differences. However, residual confounding cannot be fully excluded, particularly given the younger age of the control group. Our healthy controls represent a homogeneous population of clinically healthy individuals, whereas the long COVID cohort reflected a heterogeneous real-world population of previously hospitalized patients with varying age, vaccination status, and comorbidities. Comparison with our previously published control cohort recruited in 2021 revealed some statistically significant results (IL-6, IFN-gamma, IL-17A) (Figure S5), underscoring the influence of control group composition and temporal changes in baseline immune status [38]. In addition, recent studies suggest that baseline cytokine profiles in apparently healthy individuals may have shifted following the COVID-19 pandemic, potentially due to widespread vaccination and repeated viral exposure [39]. Therefore, the observed cytokine alterations should be interpreted cautiously and not attributed exclusively to prior SARS-CoV-2 infection.

We additionally explored the potential impact of vaccination on laboratory parameters. The observed differences in IgG levels reflect the expected humoral response to vaccination. In contrast, differences observed for other parameters were inconsistent across time points and should be interpreted with caution, as they may reflect random variation or multiple testing effects rather than biologically meaningful associations. On the other hand, we observed that vaccinated individuals showed a lower prevalence of symptoms during early follow-up (3 and 6 months), but this difference was no longer observed at 9 months. This pattern suggests that vaccination may be associated with faster symptom resolution rather than a sustained effect on long-term outcomes. A literature review by the European Centre for Disease Prevention and Control identified only limited evidence on vaccination during acute COVID-19. Notably, a single study reported a reduction in neurological complications following vaccination during the acute phase of infection, although the mechanisms remain unclear [40,41].

This study has several limitations. The long COVID cohort consisted predominantly of older men, and the sample size was relatively small. The control group included younger individuals, and samples were collected in 2025, which does not allow for complete exclusion of prior SARS-CoV-2 infection. Aging substantially affects cytokine profiles and systemic inflammatory status; therefore, residual age-related confounding cannot be fully excluded despite statistical adjustment. Further, only 12 markers were analyzed, which does not capture the full spectrum of immune responses to the virus. The next limitation was that the lack of systematic PCR testing during follow-up precluded reliable assessment of SARS-CoV-2 reinfection. Although no patients reported a confirmed episode of COVID-19 during the follow-up period, asymptomatic or unrecognized reinfections cannot be excluded.

## 4. Materials and Methods

### 4.1. Study Cohort

**Figure 5:**
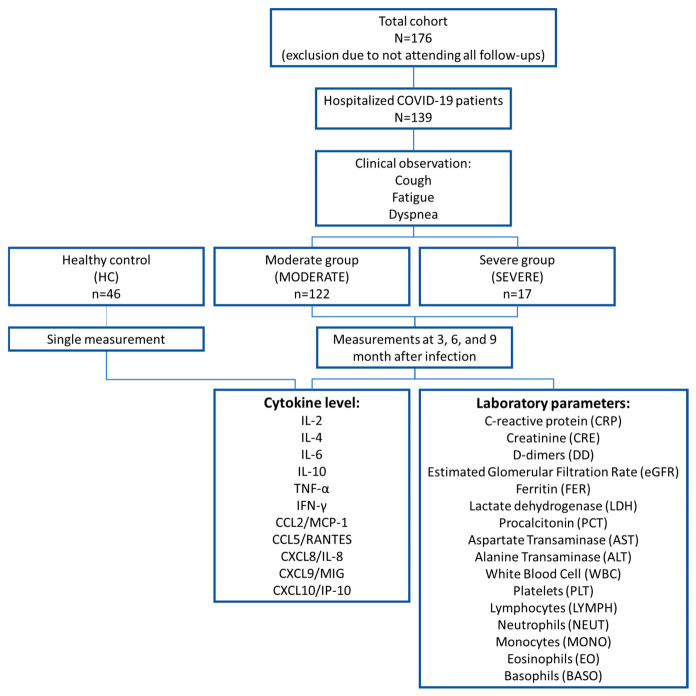
The study population, follow-up and measured parameters.

The research experiment was approved by the Ethics Committee of the Military Institute of Medicine (3/WIM/202). Separate approval was obtained for the inclusion of a HC group (KB/52/24). A total of 139 hospitalized COVID-19 patients were enrolled from the Military Institute of Medicine in Warsaw and the 7th Naval Hospital in Gdansk. The sample size was determined by patient availability during the study period rather than by a priori power analysis. The cohort included both prospective (n = 74; 46 from Gdansk and 28 from Warsaw) and retrospective observations (n = 65; 32 from Gdansk and 33 from Warsaw). Inclusion criteria included: age of 18–75 years, a positive SARS-CoV-2 PCR test on admission, and written informed consent. Exclusion criteria were: mild disease (WHO score < 4), and concomitant respiratory infections (e.g., influenza, RSV). As indicated in Figure 5, patients were classified as having moderate (n = 122) or severe disease (n = 17) according to WHO criteria. Moderate disease was defined as hospitalization with clinical signs of pneumonia and SpO_2_ > 90%, requiring supplemental oxygen but not ICU admission (WHO score 4–5). Severe disease was defined as ICU admission with pneumonia, SpO_2_ < 90%, respiratory rate > 30 breaths/min, or critical complication such as ARDS, sepsis, or septic shock (WHO score 6–7). All severe cases required mechanical ventilation. The control group consisted of healthy volunteers (n = 46) recruited in 2025 at the Regional Blood Donation and Blood Treatment Center in Warsaw. All were asymptomatic and had no evidence of prior SARS-CoV-2 exposure.

### 4.2. Sample Collection and Preparation

Patient enrollment took place between March and June 2021. Serum samples were collected during outpatient follow-up visits at 3, 6, and 9 months after hospital discharge. HCs provided a single blood sample. Peripheral blood was drawn into serum vacutainer tubes (Becton Dickinson, Amsterdam, The Netherlands), centrifuged within 2 h (2000× *g* for 15 min at room temperature), aliquoted, and frozen at −80 °C for further analysis.

### 4.3. Data Collection

Serum concentrations of CCL2/MCP-1 (CCL2), CCL5/RANTES (CCL5), CXCL8/IL-8 (CXCL8), CXCL9/MIG (CXCL9), and CXCL10/IP-10 (CXCL10) were measured using the BD Cytometric Bead Array (CBA) Human Chemokine Kit (Catalog No. 552990). IFN-γ, IL-2, IL-4, IL-6, IL-10, IL-17A, and TNF-α were quantified using the BD CBA Human Th1/Th2/Th17 Cytokine Kit (Catalog No. 560484) (Becton Dickinson, Erembodegem, Belgium). Data acquisition was performed on a CytoFLEX flow cytometer (Beckman Coulter, Brea, CA, USA) equipped with 405, 488 and 638 nm lasers. Data were analyzed using FCAP Array v3 software (Becton Dickinson, San Jose, CA, USA); values outside the linear range of the standard curve were reported as off-scale. In addition to cytokine profiling, standard inflammatory (C-reactive protein—CRP, procalcitonin—PCT, ferritin—FER, and D-dimers—DD), biochemical (creatinine—CRE, estimated glomerular filtration rate—eGFR, lactate dehydrogenase—LDH, aspartate aminotransferase—AST, and alanine aminotransferase—ALT) and hematological parameters (white blood cell count—WBC, platelets—PLT, lymphocytes—LYMPH, neutrophils—NEUT, monocytes—MONO, eosinophils—EO, and basophils—BASO) were obtained from hospital medical records. During follow-up visits, patients were also interviewed regarding the presence of cough, dyspnea, and fatigue.

### 4.4. Assessment of Data Distribution

Data distribution was assessed using the Shapiro–Wilk test, skewness, and Kurtosis. Most variables showed non-normal distributions; therefore, data are presented as medians with interquartile ranges, unless stated otherwise. A variable was considered normally distributed when the Shapiro–Wilk test yielded *p* > 0.05, skewness was within -1-1, and kurtosis was within -2-2. When comparing groups that included both normally and non-normally distributed variables, all variables were treated as non-normally distributed and non-parametric tests were applied. Analyses were performed in GraphPad Prism, version 10.6.1 (GraphPad Software, San Diego, CA, USA), and in R, version 4.5.2 (R Core Team, Vienna, Austria).

### 4.5. Statistical Analysis

Cross-sectional comparisons of cytokine concentration between groups (defined by disease severity, vaccination status, and comorbidity categories) at 3, 6, 9 months were performed using two-tailed Mann–Whitney U test or the Kruskal–Wallis test, as appropriate, followed by the Benjamini–Hochberg false discovery rate (FDR) correction. Binary clinical variables (including the presence of symptoms and comorbidities) were compared between groups using Fisher’s exact test. This approach was applied to assess associations between disease severity, vaccination status, comorbidity burden (including overall, cardiovascular and metabolic comorbidities), and symptom occurrence at each follow-up time point. Longitudinal changes in binary clinical variables (cough, fatigue, dyspnea) were assessed using Cochran’s Q test, including only patients with complete data across all follow-up time points (n = 132 for cough and fatigue, n = 131 for dyspnea). When significant, post hoc pairwise comparisons between time points were performed using McNemar’s test with Bonferroni correction. Longitudinal analysis for continuous variables across follow-up (3, 6, and 9 months) was conducted using the Friedman test with Benjamini–Hochberg FDR correction for pairwise comparisons. This non-parametric test was applied to repeated measurements obtained from the same individuals with complete data across all time points (CRP n = 135; CRE n = 135; FER n = 58; LDH n = 122; DD n = 121; eGFR n = 121; PCT n = 123; AST n = 124; ALT n = 125; WBC n = 125; PLT n = 124; LYMPH n = 125; NEUT n = 122; MONO n = 125; EO n = 125; BASO n = 125). Diagnostic performance was evaluated using receiver operating characteristic curve analysis (ROC). The area under the curve (AUC) with a 95% confidence interval was calculated to evaluate discriminatory ability between HCs and long COVID patients. Optimal cut-off values were determined using the Youden index, and sensitivity, specificity and likelihood ratios were reported. Spearman’s rank correlation coefficients were calculated to assess monotonic associations between cytokines, inflammatory markers, and hematological parameters at each follow-up time point (3, 6, and 9 months). Correlation matrices were generated separately for each visit. Two-tailed *p*-values were reported. Analyses were performed in GraphPad Prism, version 10.6.1 (GraphPad Software, San Diego, CA, USA), and in R, version 4.5.2 (R Core Team, Vienna, Austria).

## 5. Conclusions

In this longitudinal study, long COVID individuals exhibited persistent but gradually normalizing immune and hematological alterations, with most parameters approaching healthy control values by 9 months. Despite this improvement, a subset of patients continued to report symptoms, indicating that clinical recovery does not always parallel immunological resolution. Comorbidities were associated with higher symptom frequency and elevated cytokine levels, while vaccination was linked to a lower prevalence of symptoms during early follow-up. Long-term cytokine profiles showed only limited differentiation between initial disease severity groups, and IL-6 emerged as the only marker with an independent discriminatory value. However, the interpretation of cytokine alterations requires caution, as several factors—including age differences, cohort heterogeneity, vaccination status, repeated viral exposure, and temporal shifts in baseline immune profiles—may have influenced the observed patterns.

Overall, while most disturbances gradually resolve, a proportion of patients experience prolonged clinical and immunological dysregulation, underscoring the need for continued monitoring and further mechanistic research.

## Figures and Tables

**Table 1:** Characteristics of the COVID-19 and healthy control study population. The abbreviation n.d. means no data.

Variable	Long COVID	%	Healthy Control	%
Sex				
Female	51	36.7	4	8.7
Male	88	63.3	42	91.3
Age, mean ± SD, years				
Female	62.3 ± 10.4		41.0 ± 3.9	
Male	56.1 ± 12.2		34.1 ± 7.5	
BMI, mean ± SD, kg/m^2^				
Female	30.7 ± 6.1		n.d.	
Male	29.8 ± 5.2		n.d.	
Disease severity				
Moderate (WHO 4–5)	122	87.8		
Severe (WHO 6–7)	17	12.2		
Comorbidity, N, %				
Total comorbidity	116	79.9		
Hypertension	78	56.1		
Obesity	34	24.5		
Diabetes type 2	31	22.3		
Asthma	20	14.4		
Ischemic heart disease	14	10.1		
Hypothyroidism	13	9.4		
Atrial fibrillation	10	7.2		
Chronic kidney disease	7	5.0		
Chronic heart failure	7	5.0		
Prostatic hyperplasia	7	5.0		
Chronic Obstructive Pulmonary Disease	5	3.6		
Multiple sclerosis	4	2.9		
Depressive disorder	3	2.2		
Time of hospitalization, mean ± SD, days				
Total	14.4 ± 7.4			
Vaccination status, N, %				
Unvaccinated at the time of hospital admission	136	97.8		
Unvaccinated at 3-month follow-up	79	57.5		
Unvaccinated at 6-month follow-up	24	17.3		
Unvaccinated at 9-month follow-up	15	10.8		
Symptoms, N, %				
**3-month follow-up**				
Cough	39	28.7		
Fatigue	58	42.6		
Dyspnea	31	22.8		
**6-month follow-up**				
Cough	14	10.3		
Fatigue	39	28.7		
Dyspnea	10	7.3		
**9-month follow-up**				
Cough	10	7.3		
Fatigue	31	22.8		
Dyspnea	6	4.4		

**Table 2:** ROC curve analysis for individual predictive factors distinguishing healthy from long COVID individuals, including AUC, optimal cut-off values, sensitivity, specificity, likelihood ratios (LR+ and LR−), and the Youden index.

		AUC	Cut-Off	Sensitivity%	Specificity%	LR+	LR−	Youden
IL-6	3M	0.8637	6.65	81.29	82.61	4.67	0.23	63.90
	6M	0.8219	6.49	76.26	80.43	3.90	0.30	56.69
	9M	0.8639	7.44	71.94	89.13	6.62	0.31	61.07
CXCL9	3M	0.8850	93.31	82.01	86.96	6.29	0.21	68.97
	6M	0.8645	101.00	75.54	89.13	6.95	0.27	64.67
	9M	0.8777	93.37	84.17	86.96	6.45	0.18	71.13
Age		0.9602	47.50	97.83	79.14	4.69	0.03	76.97

**Table 3:** The table presents Spearman correlations meeting the criteria of ρ > 0.40 and *p* < 0.05. A green “✓” indicates that the correlation in a given group meets these conditions, whereas a red “x” denotes that no such correlation is observed.

Spearman Correlation		|ρ| > 0.4, *p* < 0.05			
		3M After COVID-19	6M After COVID-19	9M After COVID-19	HC
IL-2	TNF-alpha	✓	✗	✓	✓
IL-2	IL-4	✗	✗	✗	✓
IL-2	IL-10	✗	✗	✗	✓
IL-2	IL-17	✗	✗	✗	✓
IL-4	TNF-alpha	✗	✗	✗	✓
IL-6	IFN-gamma	✗	✗	✗	✓
CXCL8	CXCL10	✗	✓	✓	✓
CCL2	CCL5	✗	✗	✓	✗

## Data Availability

The original contributions presented in this study are included in the article/Supplementary Material. Further inquiries can be directed to the corresponding author.

## Supplementary Materials

The following supporting information can be downloaded at: https://www.mdpi.com/article/10.3390/ijms27115137/s1.

## Abbreviations

The following abbreviations are used in this manuscript:

ALT	Alanine aminotransferase
ARDS	Acute Respiratory distress syndrome
AST	Aspartate aminotransferase
AUC	Area under the receiver operating characteristic curve
BASO	Basophil count
BASO%	Basophil percentage
CRE	Creatinine
CRP	C-reactive protein
DD	D-dimers
eGFR	Estimated glomerular filtration rate
EO	Eosinophil count
EO%	Eosinophil percentage
FDR	False discovery rate
FER	Ferritin
HC	Healthy control
LDH	Lactate dehydrogenase
LYMPH	Lymphocyte count
LYMPH%	Lymphocyte percentage
MONO	Monocyte count
MONO%	Monocyte percentage
NEUT	Neutrophil count
NEUT%	Neutrophil percentage
PASC	Post-acute sequelae of COVID-19
PCT	Procalcitonin
PLT	Platelet count
ROC	Receiver operating characteristic curve
SARS-CoV-2	Severe acute respiratory syndrome coronavirus 2
WBC	Whole blood cell count
WHO	World Health Organization
