# Supplementary figures and images for: Immune Dysregulation After COVID-19: Longitudinal Analysis up to 9 Months
Source: Int J Mol Sci. 2026 Jun 5;27(11):5137. doi: 10.3390/ijms27115137 (PMC13258787; doi:10.3390/ijms27115137)

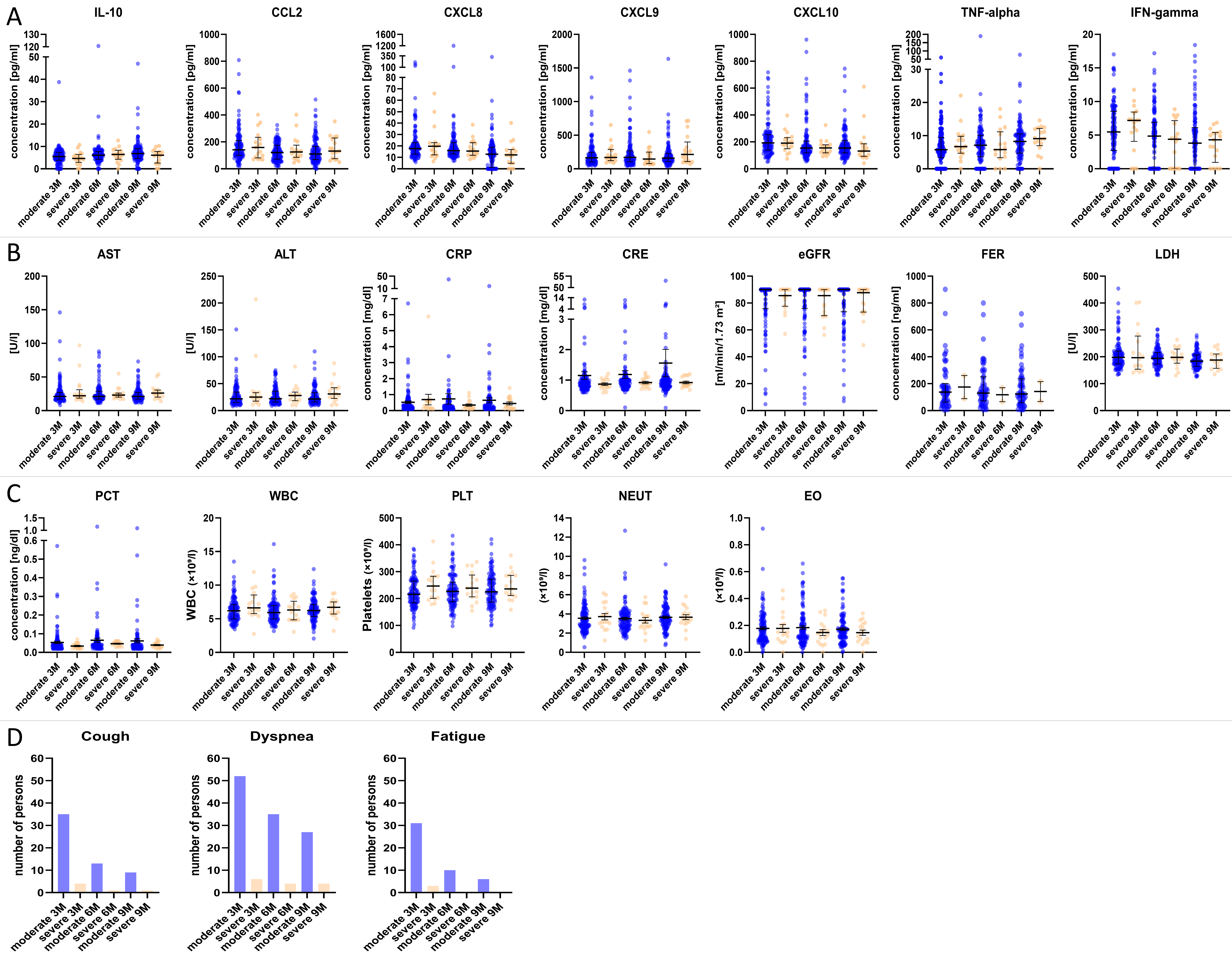

Supplement: Supplementary file 1 [file ijms-27-05137-s001.zip › S1 Figure - skompresowane S1-3 Moderate-Severe bez istotności.tif]

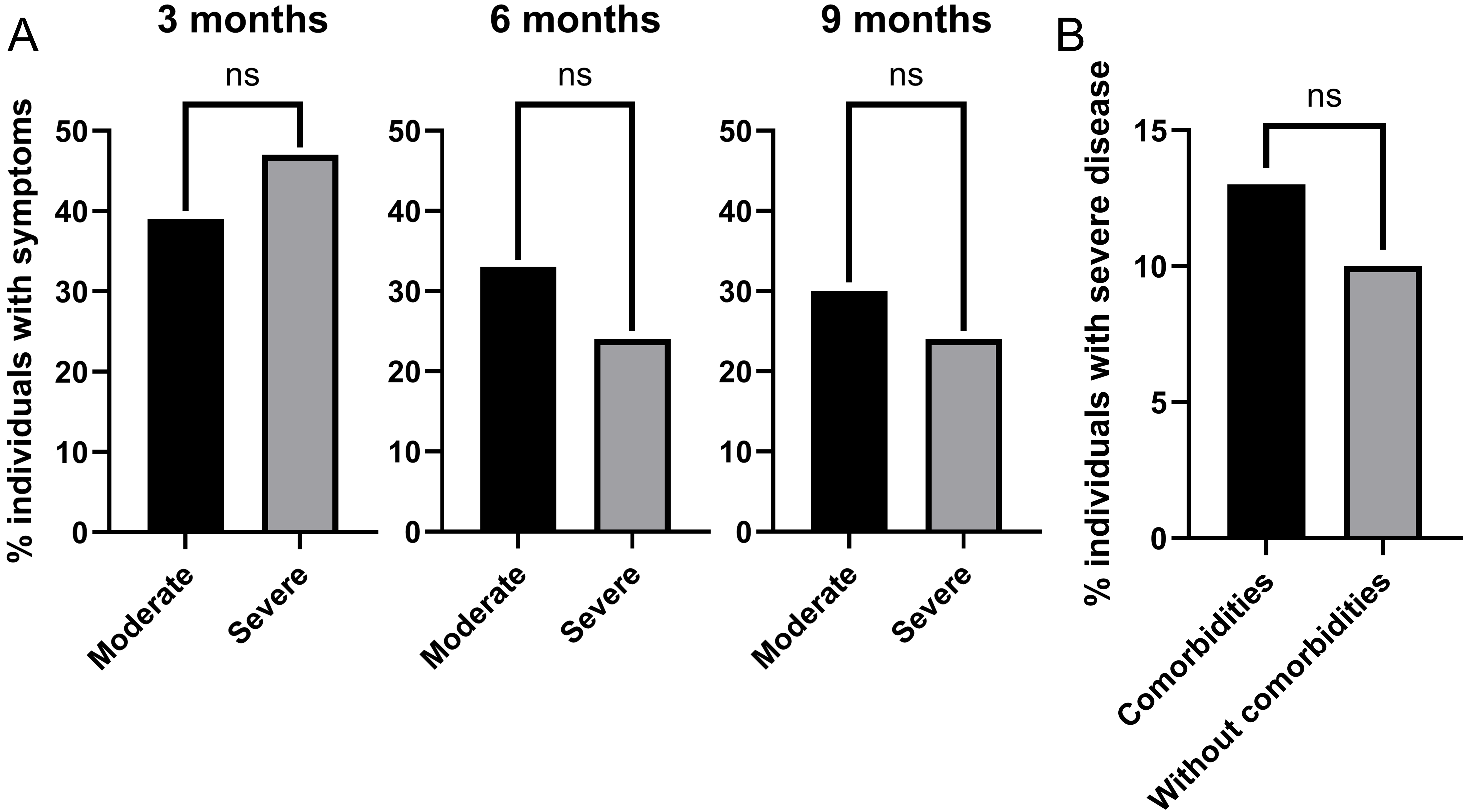

Supplement: Supplementary file 1 [file ijms-27-05137-s001.zip › S2 Figure - symptom, comorbidities vs severity.tif]

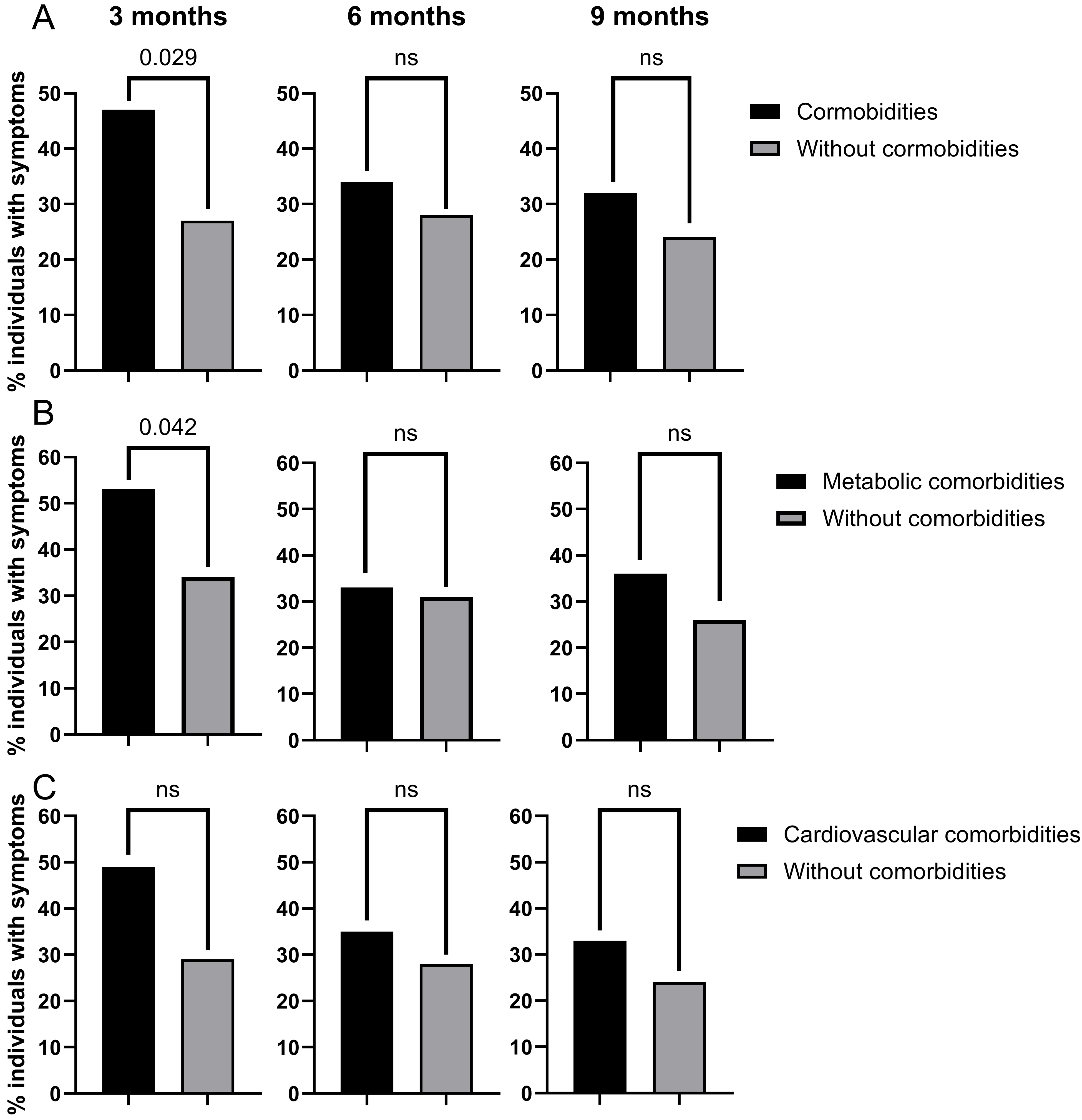

Supplement: Supplementary file 1 [file ijms-27-05137-s001.zip › S3 Figure - symptom comorbidities.tif]

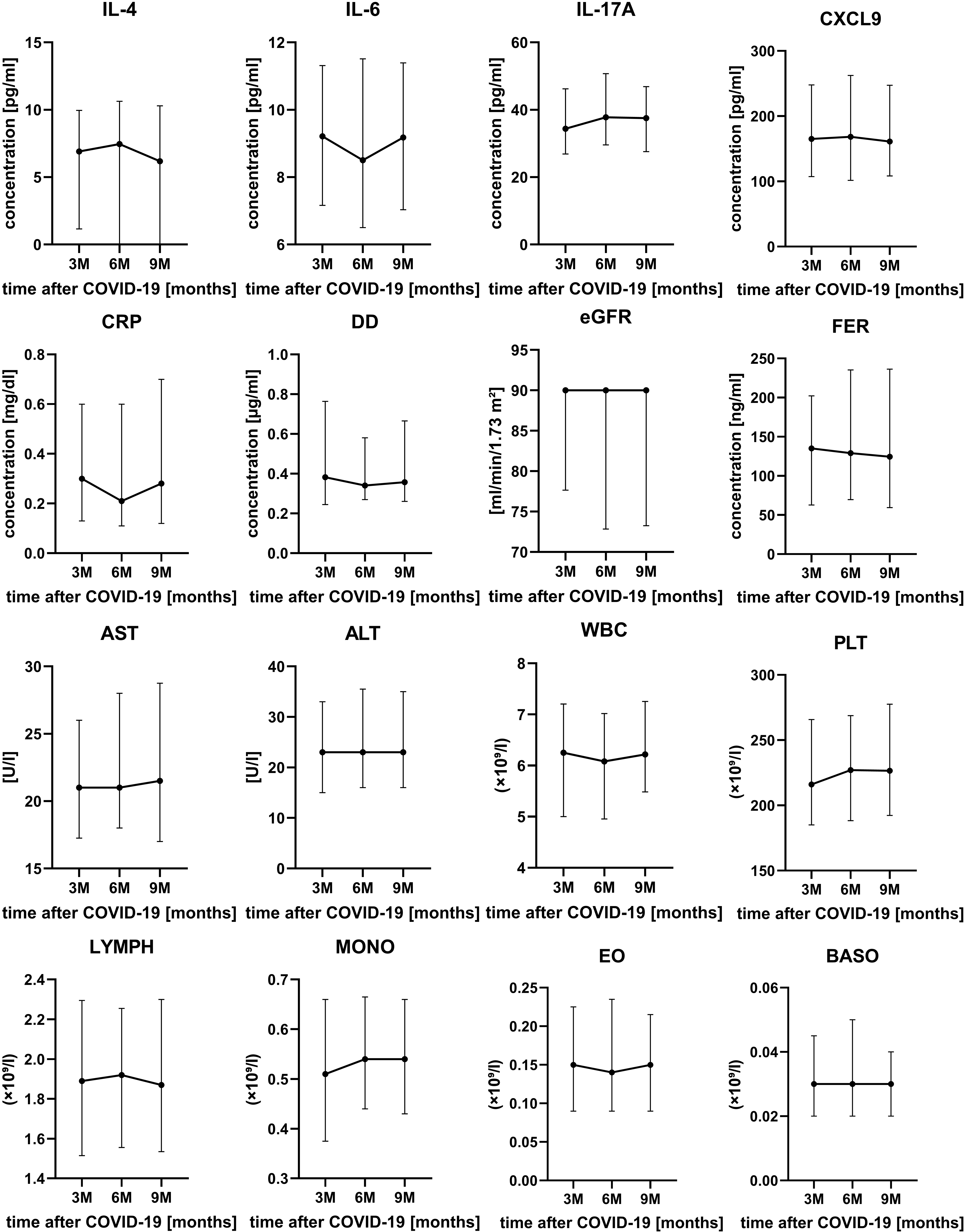

Supplement: Supplementary file 1 [file ijms-27-05137-s001.zip › S4 Figure - trendy nieistotne.tif]
